# Conservative Management of Lateral Epicondylalgia: A Review

**DOI:** 10.7759/cureus.59875

**Published:** 2024-05-08

**Authors:** Shivani R Uttamchandani, Pratik Phansopkar

**Affiliations:** 1 Musculoskeletal Physiotherapy, Ravi Nair Physiotherapy College, Datta Meghe Institute of Higher Education and Research, Wardha, IND

**Keywords:** visual analogue scale, wrist extensors, physiotherapy, extensor carpi radialis brevis muscle, lateral epicondylitis

## Abstract

A common overuse injury to the elbow is called tennis elbow or lateral epicondylitis (LE). LE is a condition that causes substantial discomfort and dysfunction in the upper extremity. Thermal and electrical agents are examples of conservative techniques. It is a significant public health concern since it frequently occurs due to occupation. It also happens in recurrent upper extremity motions including desktop use, weight training, forceful forearm movements, and repetitive vibratory movements, which are the most common activities performed by an engineer. LE leads to lateral epicondylar pain, which is irritating due to inflammation of the extensor muscle origin, and also activities of daily living are restricted. It is not only seen in players with racquet sports but then most of the males and females are affected in the fourth and fifth decades, leading to limitations in daily work, activity, and household work. Rehabilitation seems the most effective treatment yet in acute and chronic conditions if later the pain does not subside then rest, injectables, and nonsteroidal anti-inflammatory drugs are the options to be taken. Physiotherapeutic rehabilitation plays a significant role in LE.

## Introduction and background

**Figure 1:**
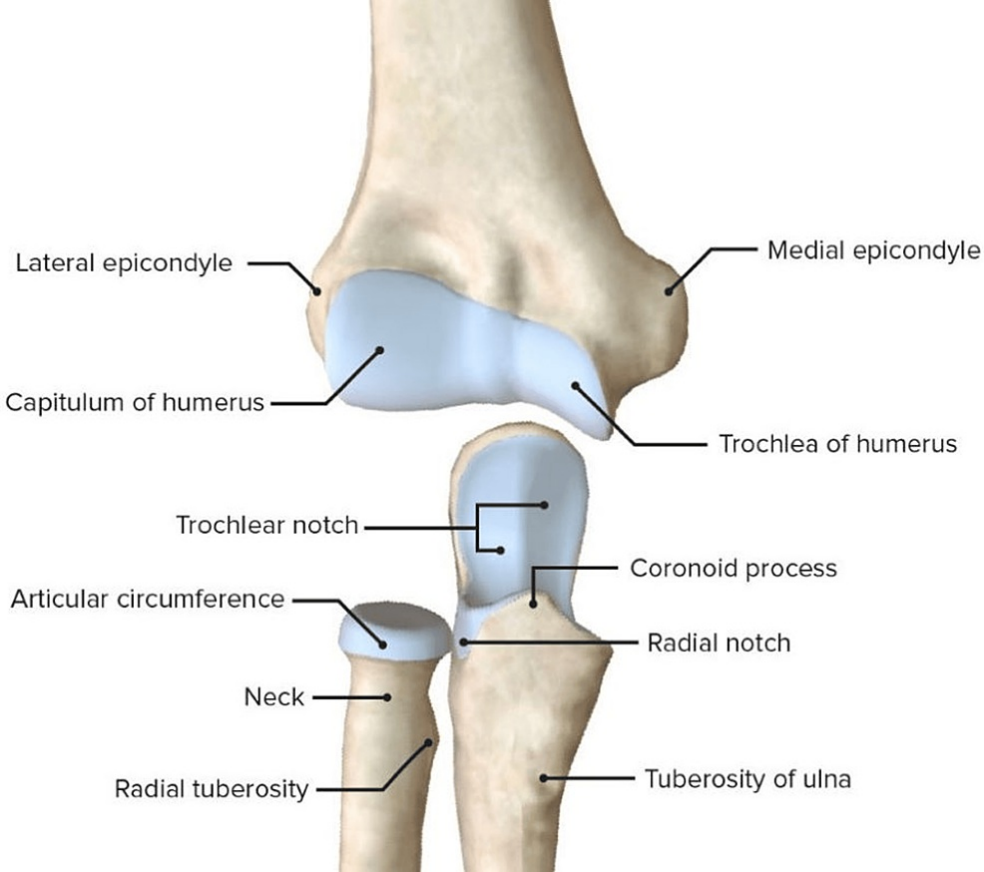
Elbow joint anatomy

Bone, cartilage, ligaments, and fluid make up your elbow joint as seen in Figure 1. Muscles and tendons assist elbow joint movement. Elbow issues occur when any of these structures are damaged or ill. Lateral epicondylitis (LE) is a common diagnosis for pain that is not caused by nerve damage or elbow instability. It is a typical issue that affects primarily women between the ages of 40 and 60; however, it can also affect men. Discomfort with lengthy wrist extension activities, discomfort during resistive wrist extension, and difficulty during rest are all frequent clinical manifestations. Histological information about angiofibroblastic tendinosis was provided [1,2]. Even if the inflammation may still be in an early stage, once symptoms appear, there is degradation and eventually fibrosis.

Between 1% and 3% of individuals worldwide suffer from LE, also known as tennis elbow, each year [3-5]. According to estimates, one million Americans get new-onset LE annually [6]. Despite its relatively high incidence, LE may result in significant discomfort and reduced functionality because of the lack of precise gold-standard treatment. However, there are numerous therapies available. LE has a significant economic impact because of interrupted working days, and some sufferers may be unable to work for many weeks [7]. According to Taylor and Hannafin, 11.7% of job-related liability claims were for medial epicondylitis and LE [8]. The current review's objective was to assess conservative physical therapy's (PT) impact and effect on LE.

## Review

Methodology

This review paper was primarily composed of journals like PubMed, Scopus, and Web of Science database, used to search for publications using regular keywords that displayed studies addressing LE, physiotherapy, recent advances, use of PowerBall Device, hand rehabilitation, or PT. The search turned up articles from many journals, including editorials, reviews, free full texts, and abstracts. After they were thoroughly examined, pertinent papers and their references were looked for in order to find other publications. Articles about strengthening devices and hand rehabilitation that included testing on 10 or more patients with diseases or injuries affecting hand function and baseline or intergroup comparisons (intervention group versus conventional PT group) were taken into consideration if they were written in English and published between 2013 and 2023. Animal studies, commentaries, case series, tales, book chapters, editorials, non-systematic reviews, and conference articles were all excluded. Duplicate publications from different databases were also ruled out.

In accordance with the protocol described by Arksey and O'Malley, study selection was carried out in two stages. One reviewer, Pratik Phansopkar, reviewed and screened titles and abstracts for relevance, and two reviewers, Shivani Uttamchandani and Pratik Phansopkar, independently evaluated the articles chosen for full-text review. To resolve disagreements and decide which articles should be included, the reviewers convened.

Epidemiology, pathogenesis, and natural history

The incidence of LE is similar in men and women, with most occurrences happening in the fourth and fifth decades of life [9]. A lack of social support, de Quervain's, carpal tunnel syndrome, oral corticosteroid usage, higher age, or body mass index (BMI), and the prevalence of rotator cuff illness are significant independent risk factors for LE [10,11]. The primary causes of LE include radial deviation, forearm supination, overtraining injuries, and strain from repeated gripping or wrist extension duties [12]. These actions can cause micro-tears, which most frequently occur at the extensor carpi radialis brevis (ECRB) tendon's origin.

Adults are prone to developing LE. It is interesting to note that only 5% to 10% of those with "tennis elbow" really play tennis [13]. The most likely cause of LE is believed to be angiofibroblastic hyperplasia, which develops as the body ages and causes vascular hyperplasia, unstructured collagen fibers in the tendons, and considerable fibroblastic activity [14,15]. Epicondylitis is believed to be caused by a recurring microtrauma that does not heal, vascular deprivation near the tendon's origin, and a weakened immunological response. The length of pain and symptoms is directly correlated with the degree of angiofibroblastic infiltration [16,17].

The proximal junction of the common extensor tendons is where people frequently complain of discomfort. Additional red flags and symptoms include shaky hands, trouble turning doorknobs, a weak grasp, and pain while clutching items [8,18]. Approximately 80% of those who experience LE report clinical improvement or remission after a year, and the majority of cases are self-limiting [19,20]. On the other side, a worse prognosis is associated with manual activities, use of the dominant arm, persistent complaints with intense baseline discomfort, and inadequate coping strategies [19]. Three to eleven percent of patients are anticipated to require surgical intervention as a result of persistent symptoms [21-23]. In a multivariate model, a researcher discovered that past injection history, earlier orthopedic surgery, employees' insurance, the existence of radial tunnel syndrome, and complaints lasting more than a year had all been indicators of surgical intervention [24].

Diagnosis

The typical patient experiences discomfort on the lateral side of the elbow, which is exacerbated by resistance to wrist extension or activities that cause the forearm to pronate. The onset of symptoms might happen quickly or gradually over time. Common concerns include writing, gardening, athletics, and keyboard use. Sports and leisure activities should also be investigated, as should aggravating factors. It is critical to ascertain whether there has ever been an elbow injury or elbow instability.

Imaging

X-rays can help identify or rule out alternate causes of lateral elbow discomfort, but they do not rule out the likelihood of LE. Magnetic resonance imaging is commonly requested by primary care practitioners. Even though T1- and T2-weighted sequences exhibit variations in signal intensity along the lateral elbow frequently, their precise importance is uncertain. In cadaver studies, degenerative changes associated with aging have been seen. Recent ultrasonography studies also revealed that 21% of the people tested had damage to the extensor origin [25,26].

Physical examination 

The purpose of a physical examination is to determine the location of pain and rule out any complicating factors. Immediately anterior to the lateral epicondyle, the lateral condylar ridge can be painful to palpate. This is especially true when resisted wrist extension is involved. While the passive range of motion is usually unaffected, active ranges of motion are typically limited by pain. To distinguish between radial tunnel syndrome and palsy of the posterior interosseous nerve, one should always palpate the route of the radial nerve, particularly throughout the whole length of the supinator muscle. Loss of active digital or thumb extension is not considered a nerve sign in the examination of LE.

One of the best methods for detecting inflammatory alterations in the tendon is ultrasound (US), especially in cases such as LE. When diagnosing lateral elbow epicondylitis, Cozen's test may be a very useful specialized test. The assessing physician instructed the subject to undertake an active wrist extension against resistance in order to complete Cozen's clinical test. The test was performed with the participant sitting in an armless chair, with the wrist in a neutral position, the shoulder slightly adducted, the forearm pronated, and the elbow extended to 90˚ [27]. A patient's lateral epicondyle must be felt with the thumb when the examiner passively pronates the forearm, flexes the wrist, and extends the elbow in Mill's test for LE. The specificity of this test is 100%, while the sensitivity is 53%. When performing Maudsley's test for LE, the patient's forearm is pronated and their elbow is flexed at a 90˚ angle. The examiner must avoid extending the patient's third finger. The soreness at the lateral epicondyle will be replicated in a positive test. It is said that this test has a 0% specificity and an 88% sensitivity [28].

Physiotherapy

At a one-month follow-up, Park et al. evaluated 31 patients with LE and found that isometric strengthening exercises reduced discomfort on the visual analogue scale (VAS) as opposed to no PT [29]. After a longer period of observation, no changes were seen (three, six, and 12 months). In 81 patients with chronic LE that lasted longer than three months, Peterson et al. discovered that PT caused a faster pain decrease at the three-month follow-up compared to those who did not undergo PT [30]. Patients, on the other hand, have not been blindfolded to the therapy, which might lead to prejudice. Coombes et al. also found that LE patients who had PT plus placebo injection had a higher incidence of full recovery at four weeks than those who had just received a placebo shot. During the one-year follow-up, the PT group took fewer analgesics and anti-inflammatory medications [31].

On 73 patients, Altun et al. evaluated the efficacy of SW vs conventional PT interventions such as thermotherapy, US, and transcutaneous electrical nerve stimulation (TENS). This study found that both groups' maximum grip strength and functionality considerably improved [32]. Applying Kinesio®® taping (KT), SW, and traditional PT, which includes cryotherapy, TENS, and an eccentric exercise program, all significantly improved pain intensity, muscle state, maximum grip strength, and functionality, according to Eraslan et al.'s comparison of the effects of these therapies [33]. Patients were randomly assigned to receive deep friction massage in conjunction with splinting and stretching, cortisone injection, or lidocaine injection in Yi et al.'s randomized controlled experiment (RCT). Deep friction massage was found to be an effective treatment for LE [34]. Patients who have not reacted to other non-operative methods, including cortisone injection, may benefit from this treatment. When isometric contractions were combined with eccentric, eccentric-concentric, and eccentric-concentric strength training for the wrist extensor muscles, the results showed that the eccentric-concentric training had the biggest effect on pain relief and functional improvement at the end of the intervention [35]. Dundar et al. examined the effects of high-intensity laser therapy (HILT) with pulse emissions at 1064 nm wavelength, 3 kW very high peak power, 360-1780 mJ/cm^22^ high fluidity level, 120-150 μ⁬s short duration, 10.5 W mean power, 10-40 Hz frequency, 0.1% work cycle, 0.5 cm beam diameter, and 0.2 cm^22^ dot size. Two additional groups were compared to this therapy: one group received a placebo laser (with the device unplugged), while the other group only used an orthosis for the whole day. The groups that had laser and orthosis showed significant improvements in grip strength, pain intensity, degree of disability, and quality of life [36].

Return to activity

Stressing that LE is a self-limited illness is the most important part of treating the condition [37,38]. Once the initial discomfort has been relieved, It could be advised for patients to return to work as soon as their symptoms allow, whether that means using wrist extension splinting or counterforce bracing. Patients are advised that discomfort is common, especially after just an exercise session, and that they can resume their sporting activities as long as the discomfort is below a bearable level and there are no symptoms of instability. 

Determining PT's effectiveness in treating LE was the goal of this review. We can state, after presenting the research that was analyzed, that PT techniques often have a positive effect on the clinical aspects of LE's remission and symptoms. The level of discomfort decreased after the application of all treatments. In every study where it was assessed, functionality increased. One of the study's limitations is that it only looked at English-language literature. One of this review's merits is that it provides an update on the various PT strategies used to treat LE. This literature analysis might be useful in future research to evaluate the validity of clinical tests performed on patients with LE and develop a new algorithm for diagnosis and assessment.

## Conclusions

Ages 40 to 60 are the most typical range of patients who experience LE. It can be annoying and last a while. A variety of therapeutic techniques, like stretching, deep friction massage, US, and TENS, have been proposed. Although many have shown short-term success, none have shown a distinct long-term benefit that goes beyond merely activity restriction, forearm rehabilitation, or even basic observation. After about a year, almost everyone experiences long-lasting relief, regardless of intervention. Although surgical therapy has been advocated, there is scant evidence that it improves the outcome. The forced immobilization and rehabilitative period that follow surgery may be the aspects of it that are most advantageous.
